# Inhibition of the Nuclear Import of Cubitus Interruptus by Roadkill in the Presence of Strong Hedgehog Signal

**DOI:** 10.1371/journal.pone.0015365

**Published:** 2010-12-15

**Authors:** Ki-Hyeon Seong, Hiroshi Akimaru, Ping Dai, Teruaki Nomura, Masahiro Okada, Shunsuke Ishii

**Affiliations:** Laboratory of Molecular Genetics, RIKEN Tsukuba Institute, Tsukuba, Japan; University of Texas MD Anderson Cancer Center, United States of America

## Abstract

Hedgehog (Hh) signalling plays an important role in various developmental processes by activating the Cubitus interruptus (Ci)/Glioblastoma (Gli) family of transcription factors. In the process of proper pattern formation, Ci activity is regulated by multiple mechanisms, including processing, trafficking, and degradation. However, it remains elusive how Ci distinctly recognizes the strong and moderate Hh signals. Roadkill (Rdx) induces Ci degradation in the anterior region of the *Drosophila* wing disc. Here, we report that Rdx inhibited Ci activity by two different mechanisms. In the region abutting the anterior/posterior boundary, which receives strong Hh signal, Rdx inhibited the nuclear import of Ci by releasing importin α3 from Ci. In this region, Rdx negatively regulated the expression of transcription factor Knot/Collier. In farther anterior regions receiving moderate levels of Hh signal, Rdx induced Ci degradation, as reported previously. Thus, two different mechanisms by which Rdx negatively regulates Ci may play an important role in the fine-tuning of Hh responses.

## Introduction

The hedgehog (Hh) family of morphogens has the crucial role for cell growth and pattern formation [1]–[4]. Furthermore, aberrant regulation in the Hh pathway leads to various diseases including cancer [5]–[9]. In *Drosophila* wing imaginal discs, posterior (P) compartment cells secret Hh protein which acts over a short range on the anterior (A) compartment cells by forming a local concentration gradient [3], [4]. Depending on the signal strength, Hh induces the expression of multiple target genes, such as *decapentaplegic* (*dpp*), *patched* (*ptc*), and *knot* (*kn*) in the A compartment cells [10]–[12].

Binding of Hh to the cell surface protein Patched (Ptc) abrogates its inhibition on another cell surface protein, Smoothened (Smo) by inducing a conformational switch [13]. This induces phosphorylation of the cytoplasmic tail of Smo by protein kinase A (PKA) and casein kinase I (CKI), leading to activation of signal transduction [14]–[17]. Hh signaling acts through the zinc finger transcription factor Cubitus interruptus (Ci) expressed in A compartment cells [2], [3]. Hh signal regulates Ci through multiple levels of regulation, including processing, nuclear-cytoplasmic partitioning, and degradation.

In A compartment cells far from the A/P compartment border which receive no Hh signal, full-length Ci (Ci-155) is phosphorylated by multiple kinases including PKA, which leads to generate a C-terminally truncated form [18]–[24]. The F-box/WD40 repeat-containing protein, Slimb, whose vertebrate homolog FWD1/βTRCP is a component of the so-called SCF (Skpl, Cdc53, and F-box) ubiquitin ligase complex, targets phosphorylated Ci-155 for ubiquitination followed by proteasome-mediated proteolysis. Ci-75 is localized in the nucleus and suppresses the expression of Hh-responsive genes such as *dpp* by acting as a repressor [25. 26]. On the other hand, in A compartment cells abutting the A/P boundary, Hh signaling prevents the processing of Ci and stimulates the activity of Ci-155 that has accumulated in the cell [18], [27].

Hh signaling also cancels the inhibitory effects of the kinesin-like protein Costal2 (Cos2) [28]–[32], and Suppressor of fused (Su(fu)) [33], [34]. Ci-155 forms complexes with the Cos2, Su(fu), and the Ser/Thr kinase Fused (Fu) that docks at microtubles. Although the tight binding of this complex to microtubles blocks the nuclear entry of Ci, activation of Fu in response to Hh signalling negates the inhibitory role of Cos2 and Su(fu) [35], [36]. After its nuclear entry, Ci-155 interacts with the transcriptional coactivator CBP to induce the transcription [37]. A biochemically uncharacterized CiA is released from the regulatory complex and begins to activate transcription, while levels of Ci155 drop [35].

The level of Ci155 is critical for correct responses to Hh, and the Ci turnover is regulated by multiple pathways. In the cells receiving very low Hh signal, Debra induces the poly-ubiquitination of phosphorylated Ci-155 in cooperation with Slimb, leading to its lysosomal degradation [38]. Debra is expressed in the band abutting the border of the Hh-responsive A compartment region of wing disc, indicating that it defines the borders of the Hh-responsive region. In the cells receiving moderate level of Hh signal, Ci-155 level is negatively regulated by Cul3-mediated degradation [39]. Roadkill (Rdx) (also called HIB) is the substrate recognition component of a Cul3 E3 ubiquitin ligase that targets Ci for degradation [40], [41]. Rdx contains the BTB and MATH domains that bind to Cul3 and the substrates, respectively [42], [43]. Rdx is induced by Hh signaling and is expressed in the Hh-responsive cells in the A compartment of wing disc, suggesting the presence of negative feedback regulation that attenuates Hh responses. Su(fu) inhibits the Rdx-mediated degradation of Ci-155 by competing with Rdx for binding to Ci [41].

Nuclear import and export of Ci-155 also play an important role to regulate Hh signaling. Hh signaling enhances the nuclear import of Ci155 by releasing Ci-155 from the complex with Cos2, Su(fu), and Fu at microtubles [28], [29], [33], [34], [44]. In fact, overexpression of Su(fu) reduces the nuclear accumulation of Ci-155. On the other hand, Su(fu) was shown to enter into the nucleus with Ci under some context [45]. Furthermore, Su(fu) inhibits the activity of Ci through a mechanism independent of Ci nuclear translocation [36].

Here, we report that Rdx inhibits Ci nuclear import in the cells receiving strong Hh signal, although it induces Ci degradation in the cells receiving the moderate levels of Hh signal. Dual mechanism of Rdx action may play an important role in fine-tuning Hh responses.

## Results

### Negative regulation of *kn* expression by Rdx in the region close to the A/P boundary

**Figure 1:**
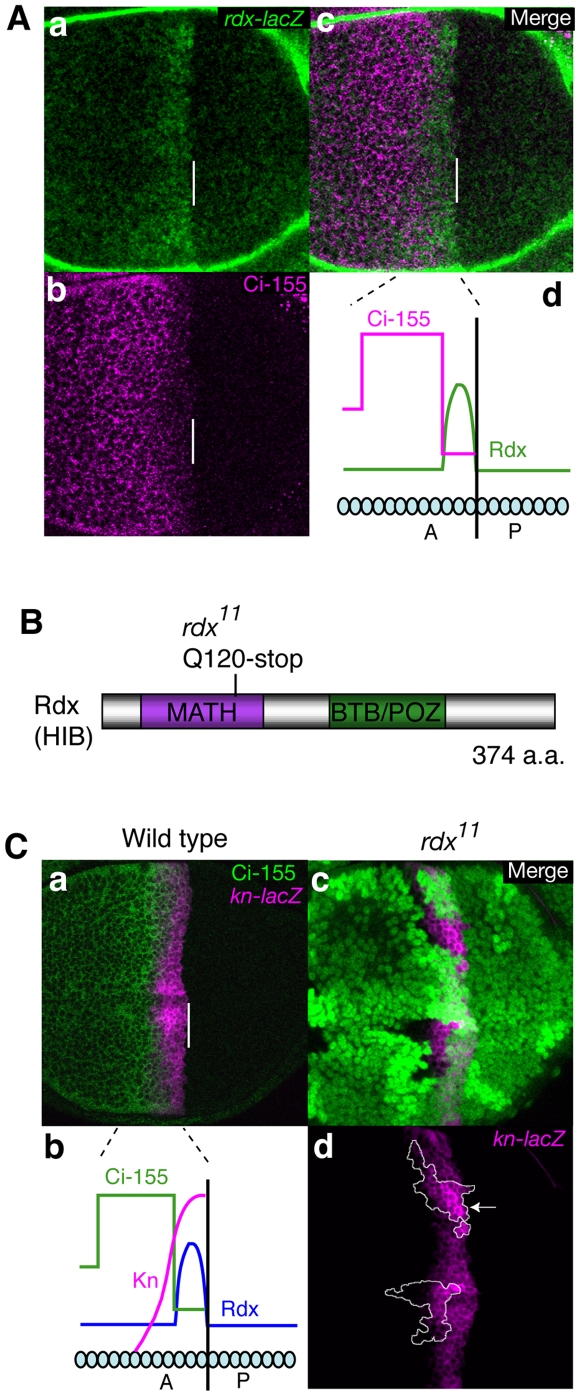
Rdx negatively regulated *kn* expression in the region close to the A/P boundary. (A) Rdx expression pattern in the wing disc. Expression of *rdx-lacZ* (green) (a) and expression of Ci-155 immunostaining (magenta) (b) are superimposed (c). Anterior is to the left; dorsal is up. Bars indicate the A/P boundary. Schematic expression pattern of Rdx is shown (d). (B) Isolation of *rdx* mutant. The domain structure of Rdx and the position of the nonsense mutation in *rdx^11^* are shown. (C) Loss of Rdx upregulated *kn*. Expression of *kn-lacZ* (magenta) and expression of Ci-155 immunostaining (green) are superimposed in (a). Schematic expression pattern of Rdx, Kn and Ci-155 is shown in (b). Clones of *rdx^11^* mutant cells marked by the absence of GFP (green) are superimposed with *kn-lacZ* expression (magenta) (c and d). Clones of *rdx^11^* are surrounded by a white line.

Using *Rdx–lacZ*, we have first monitored the precise pattern of Rdx expression in wing discs. The expression levels of Rdx were high in 2–4 cells anterior to the A/P boundary, while they were moderate in a more anterior region that is several cells-wide (Figure 1A, a and d; Figure S1) as reported previously [40], [41]. In the region abutting the A/P boundary, where Rdx is expressed at its highest level, Ci-155 levels were low (Figure 1A, b–d). In contrast, Ci-155 levels were high in the more anterior regions, which express moderate levels of Rdx (Figure 1A, b–d).

**Figure 2:**
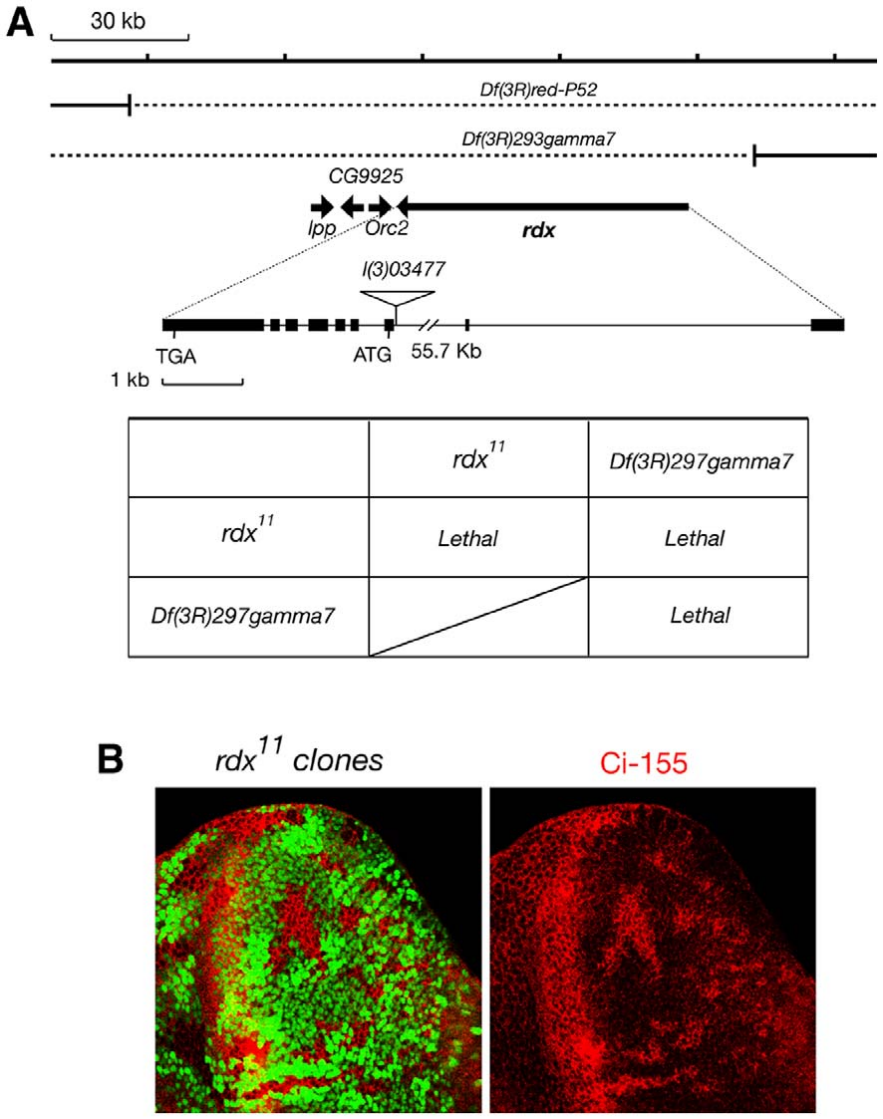
*rdx^11^* is a null mutant. (A) Classical mutant/deficiency tests were performed. The *rdx^11^/Df(3R)gamma7* heterozygotes were embryonic lethal, like the *rdx^11^/rdx^11^* and *Df(3R)gamma7/Df(3R)gamma7* homozygotes, suggesting that *rdx^11^* is a null allele. (B) Clones of *rdx^11^* mutant cells (GFP-negative) (a) in eye imaginal disc are superimposed with Ci-155 immunostaining (magenta) (b). Similar accumulation of Ci-155 was also observed in the clones of *hib^Δ6^*, which was a null mutant of *rdx* isolated by Jiang's group, and also in the clones of Df(3R)Exel6171, a deficiency line (Zhang et al., 2006). These results also support the notion that *rdx^11^* is a null mutant.

We isolated Rdx mutants using ethyl methanesulfonate treatment. One isolated allele, *rdx^11^*, had a nonsense mutation at Q120 in the MATH domain (Figure 1B). Since this mutant encoded the N-terminal 119 amino acids protein fragment, which lacks both the intact MATH and BTB domains, this mutation was thought to be a null allele. The classical mutant/deficiency test suggests that *rdx^11^* is a null allele (Figure 2A). Furthermore, accumulation of Ci-155 was observed in the clones of *rdx^11^* mutant cells (Figure 2B). Similar accumulation of Ci-155 was also observed in the clones of *hib^Δ6^*, which was a null mutant of *rdx* isolated by Jiang's group, and also in the clones of Df(3R)Exel6171, a deficiency line [41]. These results also support the notion that *rdx^11^* is a null mutant. In the *rdx^11^* homozygous clones, expression of *decapentaplegic* (*dpp*) and *patched* (*ptc*), both of which are Ci target genes [1], [2], was not up-regulated in 2–4 cells anterior to the A/P boundary (Figure S2A, B), although *dpp* expression levels were enhanced in the more anterior region, where Rdx is expressed at moderate levels (Figure S2A-b, arrow).

Kent et al. described that the *rdx* loss-of-function clones had no discernible effect on expression of *ptc* and *dpp*, although no data was shown [40]. The difference between their observations and ours could be due to the use of different alleles. Kent et al. used *rdx^5^*, which had two missense mutations, Q193H and D653V, and also *rdx^6^*, which contained a point mutation in the splice donor between exons 12 and 13. The *rdx^6^* allele generated mRNAs retaining intron 12/13 as well as wild-type mRNA. On the other hand, the *rdx^11^* allele we used had a nonsence mutation at Q120 in the MATH domain. Thus, *rdx^5^* and *rdx^6^* seem to be a hypomorphic allele, while *rdx^11^* is thought to be a null allele. Zhang et al., also reported that *ptc* was overexpressed in the *hib/rdx* mutant clones [41]. They used the *hib^Δ6^* allele, which was generated by replacing the *hib/rdx* coding region by the *white* gene, indicating this allele is a nulle allele. However, they observed *ptc* overexpression only in the presence of overexpressed Hh. So, there was no data indicating that *ptc* was overexpressed in the *hib/rdx* clones without overexpressed Hh.

In contrast to *ptc*, the expression level of *knot/collier* (*kn*/*col*), another Ci target gene in the cells abutting the A/P boundary [12], was apparently enhanced in the *rdx^11^* clones (Figure 1C and S3). Knot (Kn) is a member of the COE (Co/Olf-1/EBF) family of transcription factors [46], and controls the formation of the central intervein by inducing the expression of Blistered (the *Drosophila* homolog of serum response factor) in cooperation with Ci [12]; thus, Rdx negatively regulated the expression of *kn* in the cells close to the A/P boundary which receive the strong Hh signal.

### Rdx does not induce Ci-155 degradation in the presence of a strong Hh signal, but enhances the cytosolic retention of Ci-155

**Figure 3:**
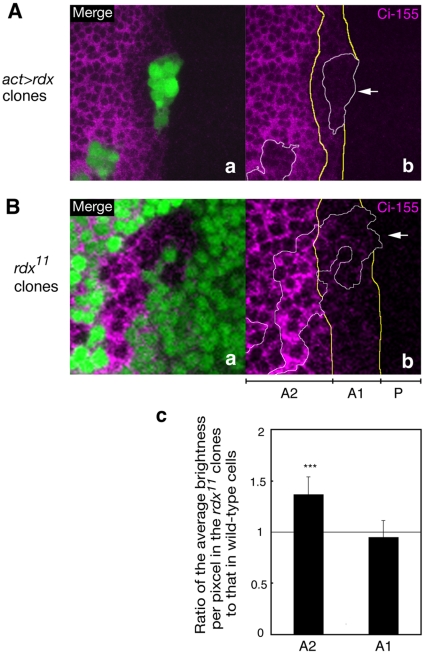
Rdx did not induce Ci-155 degradation in the region close to the A/P boundary of wing disc. (A) Overexpression of Rdx did not induce Ci-155 degradation in the region close to the A/P boundary. Clones of cells overexpressing Rdx (GFP-positive, green) (a) are superimposed with Ci-155 immunostaining (magenta) (b). Anterior is to the left; dorsal is up. Bars indicate the A/P boundary. The A/P boundary and the boundary of the regions expressing low and high levels of Ci-155 are indicated by yellow lines. Rdx overexressing clones were surrounded by white line. Arrow indicates the Rdx overexpressing clone in the region close to the A/P boundary. (B) Loss of Rdx expression did not increase the Ci-155 levels in the region close to the A/P boundary. Clones of *rdx^11^* mutant cells (GFP-negative) (a) are superimposed with Ci-155 immunostaining (magenta) (b). The A/P boundary and the boundary of the regions expressing low and high levels of Ci-155 (A1 and A2, respectively) are indicated by yellow lines. Clones of *rdx^11^* mutant were surrounded by white line. Arrow indicates the *rdx^11^* mutant clone in the region close to the A/P boundary. The ratio of average brightness per pixel in the *rdx^11^* clones to that in wild-type cells were quantified in the A1 and A2 regions, respectively, and depicted in the bar graph (c). ***, P<0.001.

Low levels of Ci-155 in 2–4 cells anterior to the A/P boundary were not further downregulated in the clones overexpressing Rdx (Figure 3A and S4A, arrow). In contrast, Ci-155 levels were reduced in cells ectopically expressing high levels of Rdx in the more anterior region, as reported previously [40], [41]. Ci-155 levels were not affected in the *rdx^11^* clones in 2–4 cells anterior to the A/P boundary (Figure 3B and S4B, arrow), although Ci-155 levels were increased by the loss of Rdx in the more anterior region, which is consistent with a previous report [41]. These results suggest that Rdx did not induce Ci degradation in the anterior narrow region abutting the A/P boundary, although it induced Ci degradation in the more anterior region.

**Figure 4:**
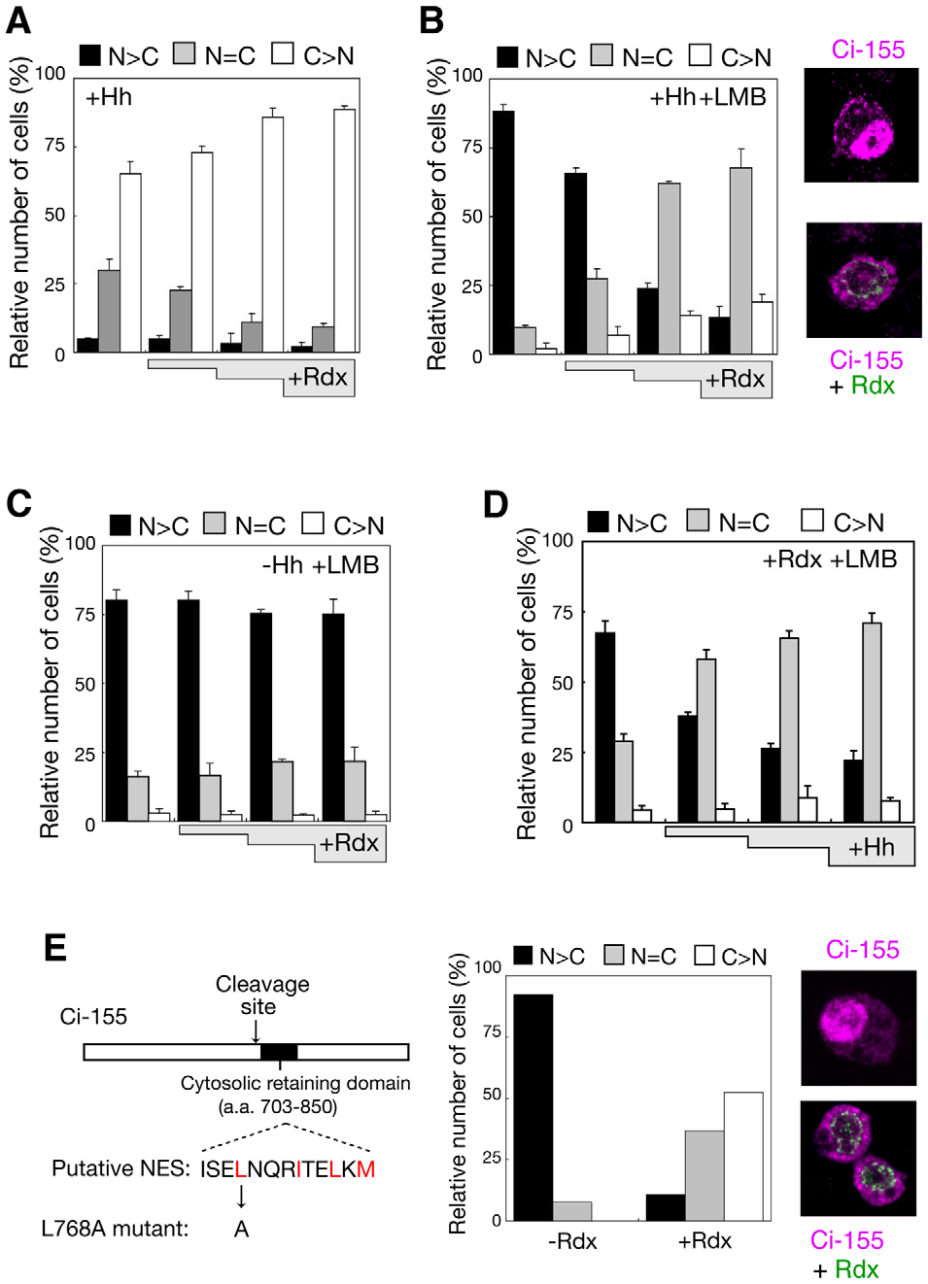
Rdx induced cytosolic accumulation of Ci-155 in the presence of a strong Hh signal. (A) Rdx retained Ci-155 in the cytosol in the presence of Hh signal. Clone-8 cells were transfected with the HA-Ci-155 expression vector and increasing amounts of the Rdx expression vector, and immunostained with the anti-HA antibody. Cells were scored by counting those in which HA-Ci was detected predominantly in nucleus (N>C), in both the nucleus and the cytoplasm (N = C), or predominantly in the cytoplasm (C>N) (total number of cells examined per one experiment: 150-300). The bar graphs depict the average percentage of cells in each category of three experiments, with SEM. (B) Effect of Rdx on Ci-155 subcellular localization in LMB-treated cells. Experiments were done as described above, except for that transfected ells were treated with LMB. On the right, typical cells immunostained with the anti-Flag and anti-HA antibodies in which HA-Ci signal was mainly detected in the nucleus (upper) or in the cytoplasm (lower). (C) Rdx did not retain Ci-155 in the cytosol in the absence of Hh signal. Experiments were done as described in (B) in the absence of Hh and in the presence of LMB. (D) Rdx more efficiently reduced nuclear Ci in the presence of higher levels of Hh. Experiments were done as described in (B) in the presence of various amounts of the Hh expression vector. (E) Effect of Rdx on the NES mutant of Ci-155. The NES mutant of Ci-155 (L768A) was generated by replacing the Leu-768 residue in the putative NES with Ala (left). Clone-8 cells were transfected with the HA-L768A expression vector and increasing amounts of the 2xFlag-Rdx expression vector, and then immunostained using the anti-HA antibody. Subcellular localization of the L768A mutant protein was examined and displayed as described in Fig. 2C (middle). Total number of cells examined was 150-200. Typical cells immunostained with the anti-Flag and anti-HA antibodies in which HA-Ci signal was mainly detected in the nucleus (upper) or in the cytoplasm (lower) are shown on the right.

To assess the role of Rdx in the presence of strong Hh signals, effect of Rdx on the subcellular localization of Ci was examined. Coexpression of Ci-155 with Rdx and Hh in Hh-responsive clone-8 cells led to an increase in the population of cells harbouring Ci-155 predominantly in the cytoplasm (Figure 4A). In addition, treatment of transfected cells with leptomycin B (LMB), an inhibitor of nuclear export, brought about an evident enhancement of the ability of Rdx to retain Ci in the cytoplasm (Figure 4B). In the absence of Hh, Rdx did not dramatically increase the population of cells harbouring Ci-155 predominantly in the cytoplasm (Figure 4C). Although Rdx induces degradation of nuclear Ci-155 in the absence of Hh [40], [41], significant amounts of nuclear Ci may be retained in the presence of LMB, so that the subcellular localization of Ci might not be affected. Rdx more efficiently reduced the population of cells harbouring Ci-155 predominantly in the nucleus in the presence of higher levels of Hh (Figure 4D). Similar results were observed using a Ci mutant, in which the nuclear export sequence was mutated (Figure 4E); thus, in the presence of strong Hh signal, Rdx enhanced the cytosolic retention of Ci-155.

### Rdx inhibits the nuclear import of Ci-155 only in the presence of strong Hh signal

**Figure 5:**
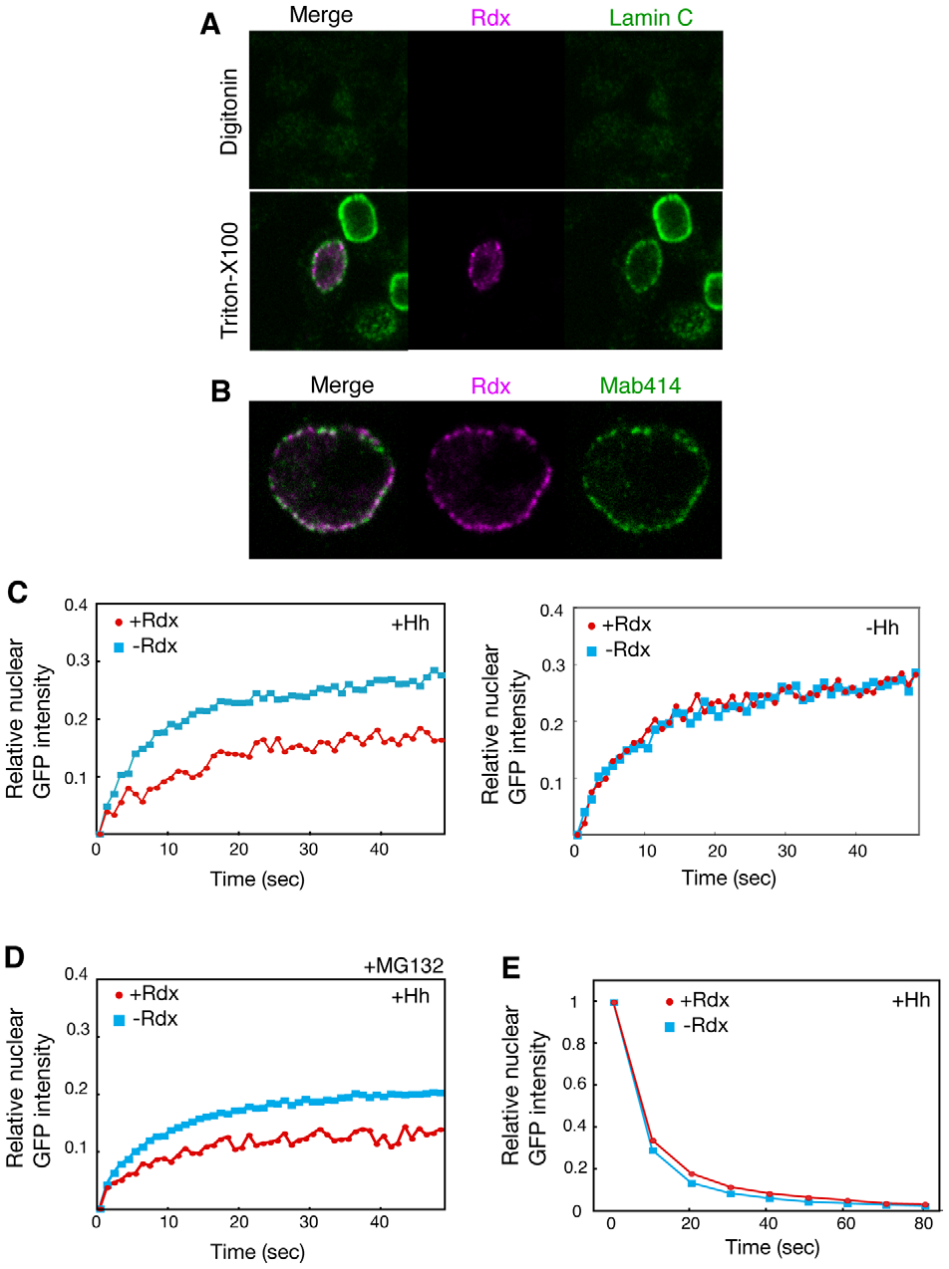
Rdx inhibited the nuclear import of Ci-155. (A, B) Localization of Rdx at the nuclear pores. Clone-8 cells were transfected with the HA-Rdx expression vector, stained with anti-HA antibody (magenta), anti-lamin antibody (green) (A), or monoclonal antibody 414 (green) (B), after treatment with digitonin (upper in A) or Triton X-100 (lower in A and B), and were then analyzed by confocal microscopy. (C) Rdx inhibited the nuclear import of Ci-155 only in the presence of a strong Hh signal. FRAP experiments were performed using clone-8 cells transfected with the Ci-GFP and Hh expression vector, in the presence or absence of the Rdx expression plasmid (upper). The average of multiple experiments at each time point (n = 8 for –Rdx and n = 6 for +Rdx) is shown. Similar experiments were performed, with the exception of the absence of the Hh expression vector (lower). The average of multiple experiments at each time point (n = 8 for –Rdx and n = 10 for +Rdx) is shown. (D) Rdx inhibited the nuclear import of Ci-155 even in the presence of MG132. FRAP experiments were performed in the presence of the Hh expression palsmid as described above. Transfected cells were treated with MG132 (50 µM) for 5 h before harvesting the cells. (E) Rdx did not affect the nuclear export of Ci-155. FLIP experiments were performed using clone-8 cells transfected with the Ci-GFP and Hh expression vector, in the presence or absence of the Rdx expression plasmid. The decrease in nuclear Ci-GFP signal after cytosolic photobleaching was assessed, to monitor nuclear export. The average of multiple experiments at each time point (n = 6 for both –Rdx and +Rdx) is shown.

To understand the mechanism by which Rdx enhanced the cytosolic retention of Ci-155, we have examined the subcellular localization of Rdx in clone-8 cells. Rdx was detected in the nuclear periphery region in an immunostaining experiment using Triton X-100-treated clone-8 cells (Figure 5A, lower). However, Rdx was not detected in the cells treated with digitonin, which can permeabilize the plasma membrane but not the nuclear membrane (Figure 5A, upper), indicating that Rdx is localized at the inside of the nuclear membrane. Rdx signal was detected in speckle-like structures located on the inside of the nuclear membrane, and overlapped with nuclear pores immunostained using the monoclonal antibody 414 (Figure 5B), which recognizes a common nucleoporin epitope [47], [48]. This suggests that Rdx may regulate nuclear import of Ci.

To test this hypothesis, fluorescence recovery after photo bleaching (FRAP) experiments were performed using Ci-155 fused to GFP (Ci-GFP). After bleaching Ci-GFP fluorescence in the nucleus, compartmental equilibration (which occurs via nuclear import) was measured by time-lapse fluorescence microscopy. When Ci-GFP was coexpressed with Hh in clone-8 cells, the rate of nuclear import of Ci-GFP was 2.4±0.5 s^−1^/100 (Figure 5C, left and Figure S5A, B). Coexpression of Ci-GFP with Rdx apparently reduced the rate of nuclear import of Ci-GFP, and the rate of nuclear import was 1.9±0.5 s^−1^/100. The significant effect of Rdx on the amount of nuclear Ci-GFP was observed within 10 sec after photpbleaching, indicating that this was not due to the Rdx-induced degradation of Ci, because the turnover rate of Ci is not so rapid. Thus, Rdx decreased the rate of nuclear import of Ci-GFP in the presence of strong Hh signal (P<0.05). When the FRAP experiments were performed in the absence of the Hh expression vector, the rate of nuclear import of Ci-GFP was similar in the presence and absence of Rdx (Figure 5C, right and Figure S5C), indicating that Rdx did not affect the nuclear import of Ci-GFP; thus, Rdx negatively regulates the nuclear import of Ci-155, but only in the presence of a strong Hh signal. To further confirm that the effect of Rdx in the FRAP experiment is not due to the Rdx-induced degradation of Ci-155, similar experiments were performed in the presence of MG132, an inhibitor of proteasome. Even in the presence of MG132, Rdx decreased the rate of nuclear import of Ci-GFP in the presence of strong Hh signal (P<0.05) (Figure 5D). The decrease in the saturated level of relative nuclear Ci-GFP intensity may be due to increased levels of cytosolic Ci-GFP by MG132. To examine the effect of Rdx on the nuclear export of Ci-155, fluorescence loss in photobleaching (FLIP) was used. Similar Ci-GFP nuclear export patterns were observed in the presence or absence of Rdx (Figure 5E and Figure S6), indicating that Rdx does not affect the nuclear export of Ci-155.

### Rdx inhibits the nuclear import of Ci-155 by competing with importin α3 for binding to Ci-155

**Figure 6:**
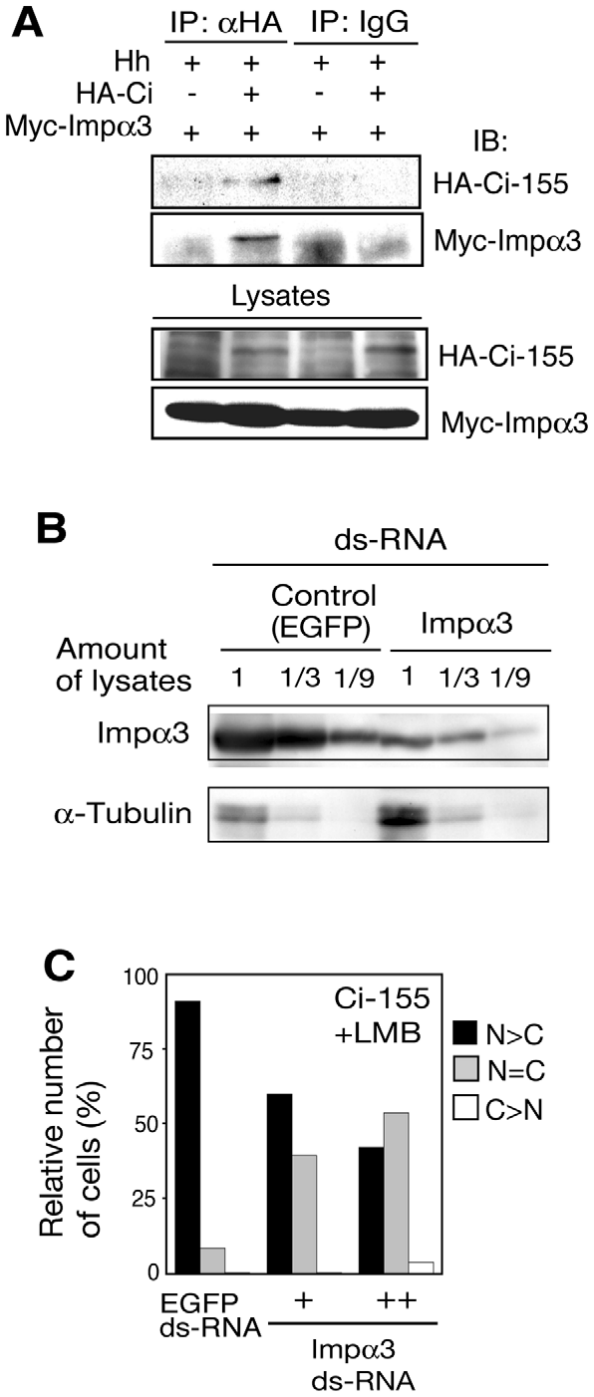
Importin α3 mediated the nuclear import of Ci-155. (A) Binding of importin α3 to Ci-155. Clone-8 cells were transfected with the Myc-importin α3, HA-Ci-155 and Hh expression plasmids. Lysates from the transfected cells were immunoprecipitated with anti-HA antibody or control IgG, and the immunocomplexes were analyzed by western blot using an anti-HA or anti-Myc antibody. (B) Downregulation of importin α3 by RNAi. Clone-8 cells were treated with importin α3 or control (EGFP) dsRNA. The indicated amounts of cell lysates were analyzed by SDS–PAGE, followed by western blotting using anti-importin α3 or anti-α-tubulin antibody. (C) Role of importin α3 in the nuclear import of Ci-155. Clone-8 cells were treated with importin α3 or control (EGFP) ds-RNA, and were then transfected with the HA-Ci-155 expression vector. Cells were treated with LMB and the subcellular localization of Ci-155 was examined as described in Fig. 2C (number of cells examined: 200–300).

When we screened the Ci-binding proteins using a yeast two-hybrid assay, we have also identified importin α3, in addition to Rdx (see Materials and methods). Furthermore, another group also identified importin α3 in the two-hybrid screening using Ci as a bait [49]. Importin α3 mediates the nuclear import of multiple proteins [50]. To examine whether importin α3 interacts with Ci *in vivo*, co-immunoprecipitation assays were performed. Importin α3 was co-immunoprecipitated with Ci-155 in lysates of clone-8 cells (Figure 6A).

To investigate whether knock down of importin α3 affects the nuclear import of Ci, we first tried a down-regulation of importin α3 using double-starnded RNA (ds-RNA). Transfection of the importin α3 ds-RNA into clone-8 cells decresaed the levels of importin α3 to about 1/10 of the control (Figure 6B). Knock down of importin α3 levels in clone-8 cells by RNAi in the presence of LMB led to a decrease in the population of cells harbouring Ci-155 predominantly in the nucleus (Figure 6C). These results indcate that importin α3 binds to Ci and acts as a nuclear import receptor for Ci.

**Figure 7:**
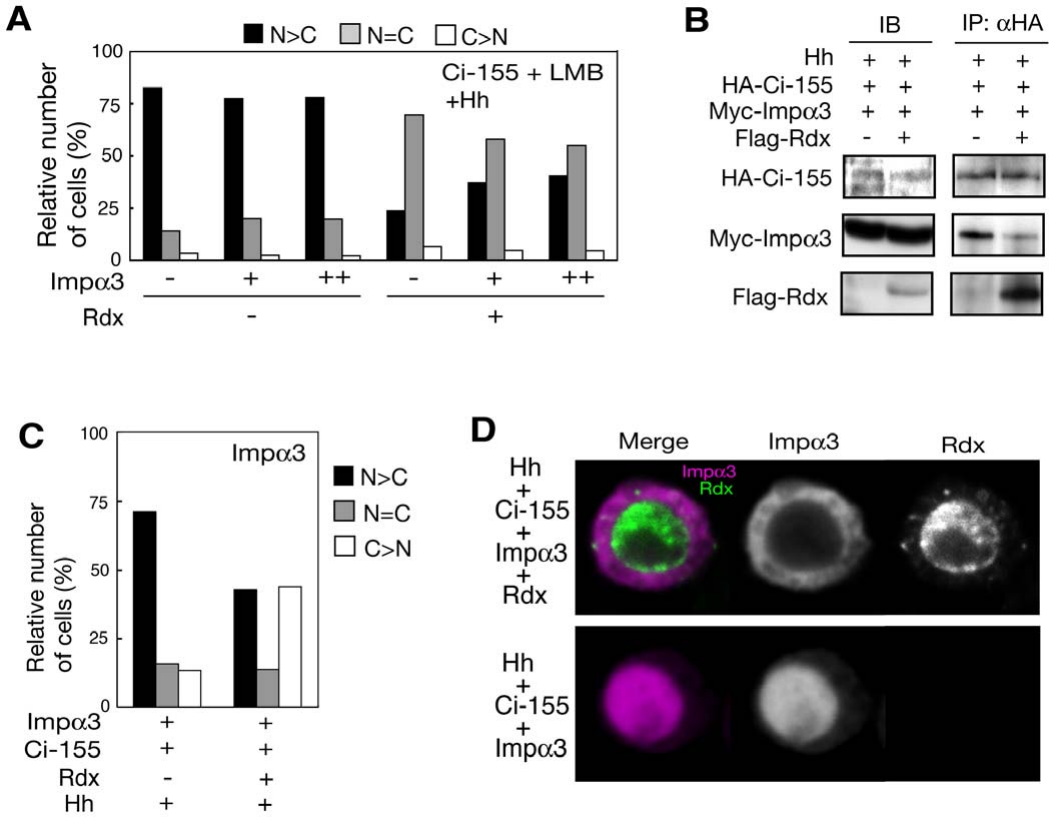
Rdx inhibited the nuclear import of Ci-155 by competing with importin α3 for binding to Ci-155. (A) Importin α3 counteracted the Rdx-mediated inhibition of nuclear import of Ci-155. HA-Ci-155 was expressed in clone-8 cells, with or without Rdx and varying amounts of importin α3. Cells were treated with LMB and the subcellular localization of Ci-155 was examined as described above (number of cells examined: 200–250). (B) Rdx competed with importin α3 for binding to Ci-155. Clone-8 cells were transfected with the HA-Ci-155, Myc-importin α3 or Hh expression plasmids, in the presence or absence of the Rdx expression plasmid. Lysates from the transfected cells were immunoprecipitated with anti-HA antibody, and the immunocomplexes were analyzed by western blotting to detect the proteins indicated on the left. (C and D) Rdx inhibited the nuclear localization of importin α3. Clone-8 cells were transfected with the Ci-155 and Myc-importin α3 expression plasmids, in the presence or absence of the 2xFlag-Rdx expression vector. Cells were immunostained with the anti-Myc antibody. Cells were scored as described in Fig. 2 (number of cells examined: 80–100) (C). Typical cells immunostained with the anti-Myc and anti-Flag antibodies are shown (D).

Importin α3 overexpression did not enhance the nuclear accumulation of Ci-155 (Figure 7A, left). This may be due to the presence of saturated amount of endogenous importin α3 in clone-8 cells. When importin α3 was coexpressed with Rdx, however, the Rdx-dependent inhibition of the nuclear import of Ci-155 was counteracted by importin α3 (Figure 7A, right). These results suggest a competitive action of Rdx and importin α3. To directly assess the competitive action of Rdx and importin α3, the effect of Rdx on the Ci-importin α3 interaction was examined. Importin α3 was co-immunoprecipitated with C-155 in the absence of exogenously expressed Rdx, whereas this interaction was abrogated by overexpressed Rdx (Figure 7B). Thus, Rdx competes with importin α3 for binding to Ci-155. These results suggest that Rdx may inhibit the nuclear import of Ci by competing with importin α3 in the presence of a strong Hh signal. If Rdx inhibits the nuclear entry of Ci-155 by competing with importin α3, furthermore, Rdx would also block the nuclear entry of importin α3 in the presence of Ci-155. To test this, we have also examined whether Rdx affects the nuclear entry of importin α3 itself. Rdx also inhibited the nuclear accumulation of importin α3 when importin α3 was coexpressed with Ci-155 (Figure 7C and 7D).

## Discussion

**Figure 8:**
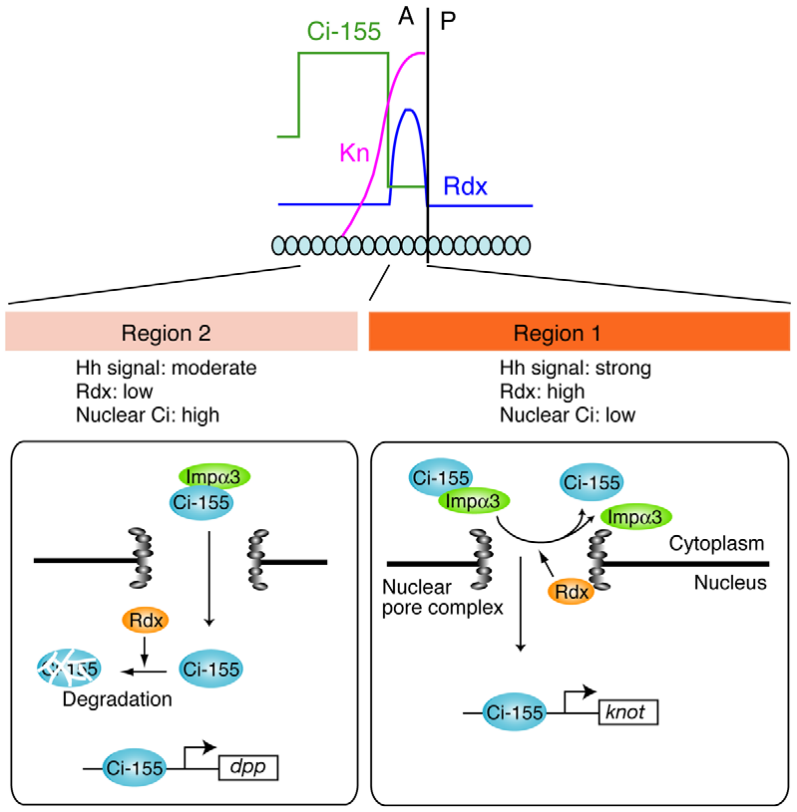
Model for the switching of the dual function of Rdx depending on Hh signal strength. In region 1 abutting the A/P boundary, which receives strong Hh signal, the Rdx levels are high. In this region, Rdx inhibits the nuclear import of Ci-155 by competing with importin α3 for binding to Ci-155 at the nuclear pore complex. This maintains the nuclear Ci-155 at low levels. Low levels of nuclear Ci-155 selectively activate the transcription of *kn*. Low levels of nuclear Ci-155 may be central to the selective induction of specific Hh target genes, such as *kn*. On the other hand, in region 2 distant from the A/P boundary, which exhibits moderate levels of Hh signal, the Rdx levels are moderate. In this region, the nuclear import of Ci-155 is allowded, which maintains the nuclear Ci-155 at relatively high levels; however, the nuclear Ci-155 level is negatively regulated by Rdx via a proteasome-dependent degradation. The relationship between Hh signal, Rdx and Ci is shown below. Strong Hh signal induces Rdx expression, which inhibits the nuclear entry of Ci-155.

In the region close to the A/P boundary, strong Hh signal induces the high level of Rdx expression, and Rdx inhibits the nuclear import of Ci-155 (Figure 8). In the more anterior region, moderate level of Hh signal induces the moderate level of Rdx, and Rdx induces the proteasome-dependent degradation of Ci-155. Why Rdx uses different mechanism to inhibit Ci activity depending on the Hh signal strength? In the region close to the A/P boundary, Ci level is low and a limited number of Ci target genes, such as *kn*, is regulated by Rdx. Kn is a member of the COE (Co/Olf-1/EBF) family of transcription factors [46], and controls the formation of the central intervein by inducing the expression of Blistered in cooperation with Ci [12]. To selectively regulate a limited number of target genes, maintaining nuclear Ci at low levels may be important. To do this, inhibition of nuclear import could be more efficient than degradation in the nucleus. Thus, switching the dual function of Rdx depending on the Hh signal strength may exhibit the elegant regulatory system in fine-tuning Hh response.

Ci-155 binds to importin α3, which forms a heterodimer with importin β [51], suggesting that the ternary complex containing Ci-155 and importin α3/β is transported into the nucleus via the nuclear pore complex (NPC). The NPC that is composed of about 30 different proteins termed nucleoporins (nups) is a cylindrical structure [52]. The nups containing phenylalanine-glycine (FG) repeats make up about half the mass of the NPC [52] and the hydrophobic FG repeats interact weakly with each other to form a “selective sieve” [53]. The hydrophobic nature of importins enables them to enter the sieve and travel via diffusion along an affinity gradient of nup-binding sites, encountering nups of increasing affinity during their translocation. After translocation through the NPC, the Ci-155-importin α3/β complex encounters RanGTP, leading to cargo association and release for termination of the transport cycle. Rdx is colocalized with nups at the NPC, suggesting that it binds to Ci-155 during the Ci-155-importin α3/β complex travels in the FG repeats sieve. Binding of Rdx to Ci-155 would release importin α3 from Ci-155, resulting to exclude Ci-155 from the FG repeats sieve. Thus, localization of Rdx at the NPC is essential to block the nuclear import of Ci-155. Although majority of Rdx molecules are at the NPC in clone-8 cells, Rdx was also detected in the specific region of nucleoplasm. Therefore, Rdx localized in the nucleoplasm may lead to the proteasome-dependent degradation of Ci-155, while Rdx at the NPC may inhibit the nuclear import of Ci-155, although we cannot exclude the possibility that Rdx at the NPC regulates both the degradation and nuclear import of Ci-155.

Zhang et al. showed that Su(fu) competes with Rdx for binding to Ci-155, and negatively regulates the Rdx-dependent degradation of nuclear Ci-155 [41]. Su(fu) was previously shown to retain Ci-155 in the cytoplasm [33], [34]. However, we have observed that in the presence of Rdx, Su(fu) enhances the nuclear entry of Ci-155 (data not shown). This suggests that Su(fu) could enhances the nuclear entry of Ci-155 by competing with Rdx for binding to Ci-155. Further analyses will be required for understanding the role of Su(fu) in the Rdx-dependent regulation of Ci-155.

## Materials and Methods

### Yeast two-hybrid screening

The yeast two-hybrid assay was carried out according to the methods described by Vojtek et al. [54]. The "bait" was a Ci fragment (amino acids 1–441) fused to the LexA DNA–binding domain. The *Drosophila* whole adult cDNA library (Clontech) was used. Among the 81 clones isolated, 32 clones were derived from the *rdx* gene, while 7 clones enclosed *importin α3*.

### Isolation of *rdx* mutants

*w^1118^* males were fed 25 mM ethyl methanesulfonate (EMS) and then mated with *w; Pr, Dr/TM6B, Tb, Sb* virgin females. The *TM6, Tb* balanced F1 males were used to cross single male lines with virgin females of genotype *w; Df(3R)red-P52/TM3, Ser, Sb*. Eleven thousand four hundred mutagenized third chromosomes were used for the screening and 20 recessive lethal candidate lines were established. Candidate lines were also crossed with *meiP19^M2^/TM6B, Tb, Sb* (*rdx* enhancer trap line and recessive lethal mutant of rdx) [41] and *Df(3R)293gamma^7^/TM6B, Tb, Sb* for complementation tests, and seven lines were identified as recessive lethal mutants of the rdx locus. These lines were backcrossed to *w^1118^* six times.

### Plasmid construction

The full-length *rdx* cDNA, which encodes the B isoform of CG9924, was cloned from the wh *Drosophila* ole adult cDNA library (Clontech). The importin α3 cDNA was obtained from S. Cotterill [55]. The plasmids to express the following proteins were constructed using a vector containing the *Drosophila act5C* promoter: Hh, HA-Ci-155, 3xFlag-Ci-155, HA-Ci-155 (L768A), HA-Rdx, 2xFlag-Rdx and Myc-Importin α3. In these constructs, the HA-, Flag-, or Myc-tag was placed at N-terminus. The mutant of the nuclear export sequence (NES) of Ci (L768A) was constructed using a PCR-based method.

### Fly stocks and generation of marked clones

Flies were reared at 25°C on a standard yeast/cornmeal/glucose/agar medium. The following stocks were used in this study: *rdx^11^*, *rdx-lacZ* (named *P{PZ}mei-P19^03477^*) [40], *dpp-lacZ^BS3.0^* [56], *ptc-lacZ* [11], *kn-lacZ* (designated as *kn^Mel^701-1991-lacZ*, obtained from S. B. Carroll) [57], *UAS-rdx*, *UAS-hh* [58], *UAS-Su(fu)* (obtained from D. Busson) [59], *UAS-Su(fu)IR* (obtained from R. A. Holmgren) [45] and *MS1096-GAL4* [58]. Somatic mutant clones and GAL4/UAS-mediated overexpression clones were generated by FLP/FRT-mediated mitotic recombination and Flp-out technique, respectively [60], [61]. Each clone was generated by heat shock for 60 min at 37°C 48–72 h after egg laying.

### UAS-rdx construct and germ line transformation

The 2.1 Kb full-length *rdx* cDNA was cloned between the EcoRI and NotI sites of the pUAST vector [62]. This construct was co-injected with the Δ2–3 helper plasmid into the *w^1118^* host line.

### Genotypes

The flies used in this study had the following genotypes:

*rdx-lacZ/+* (Figure1A and S1A); *hh-GAL4 UAS-GFP/rdx-lacZ* (Figure S1B); *kn-lacZ/+* (Figure 1C-a); *kn-lacZ/hs-flp; 82BFRT rdx^11^/82BFRT ubi-GFP* (Figure 1C-c, d and S3); *hs-flp/+; AyGAL4 UAS-GFP/+; UAS-rdx/+* (Figure 3A and S4A); *hs-flp/+; 82BFRT rdx^11^/82BFRT ubi-GFP* (Figure 3B and S4B); *hs-flp/+; dpp-lacZ^BS3.0^/+; 82BFRT rdx^11^/82BFRT ubi-GFP* (Figure S2A); *hs-flp/+; 82BFRT rdx^11^/82BFRT ubi-GFP* (Figure S2B).

### Immunohistochemistry

Imaginal discs from *Drosophila* third instar larvae were fixed and stained using standard techniques. Primary antibodies used in this study were: rat anti-Ci (2A1) (diluted 1∶50, gift from R. Holmgren), rabbit anti-β-galactosidase (1∶500, CAPPEL), mouse anti-Ptc (1∶50, gift from I. Guerrero), mouse anti-En (4D9) (1∶50, Developmental Studies Hybridoma Bank (DSHB)), rat anti-HA 3F10 (1∶200, Roth), mouse anti-Flag M2 (1∶200, Sigma) and mouse anti-Myc (1∶200, MBL). Secondary antibodies coupled to Alexa-488 (Molecular Probes), Cy3 and Cy5 (Jackson) were used at 1∶250. Immunofluorescent stainings were visualized using a Zeiss confocal laser microscope 510.

### Subcellular localization of Ci-155 *and* Importin α3

Using Cellfectin (Invitrogen), clone-8 cells (2×10^6^ cells) in 6 well dish were transfected with a mixture of the plasmid to express the following proteins: for Figure 4A, 4B, and 4C, HA-Ci-155 (500 ng), Hh (500 ng), and 2xFlag-Rdx (0, 50, 250, or 500 ng); for Figure 4D, HA-Ci-155 (500 ng), Hh (0, 100, 250, or 500 ng), and 2xFlag-Rdx (500 ng); for Figure 4E, HA-Ci-155 (L768A) (500 ng), Hh (500 ng), and 2xFlag-Rdx (0 or 500 ng); for Figure 6C, HA-Ci-155 (500 ng), Hh (500 ng), and importin α3 ds-RNA (5 or 10 µg) or control ds-RNA (10 µg); for Figure 7A, HA-Ci-155 (500 ng), Hh (500 ng), 2xFlag-Rdx (0 or 500 ng), and Myc-Importin α3 (0, 250, or 500 ng); for Figure 7B, HA-Ci-155 (500 ng), Hh (500 ng), 2xFlag-Rdx (0 or 500 ng), and Myc-Importin α3 (500 ng). The total amount of DNA was adjusted to 2 µg by adding the empty vector. In some cases, transfected cells were treated with leptomycin B (LMB) (10 ng/ml) for 4 h before fixation. Forty-eight hours after transfection, transfected cells were fixed with 4% paraformaldehyde/PBS for 30 min and then washed with PBS containing 0.3% Triton X-100 (PBTx) for 10 min. Samples were incubated with 10% goat serum/PBTx for 1 h, and then incubated with the anti-HA 3F10 antibody. After washing with PBTx, samples were incubated with Cy3-conjugated rat IgG. Cells were mounted with Gel/Mount (Biomeda) and examined by fluorescence microscopy (Zeiss).

### Fluorescence Recovery after Photobleaching (FRAP) and Fluorescence Loss in Photobleaching (FLIP) analysis

Clone-8 cells (2×10^6^ cells/2.5 cm dish) were transfected with a mixture of the following plasmids: for Figure 5C (left), 5D and S5B, act5c-hh (500 ng), UAS-Ci-GFP (23) (200 ng), act5C-GAL4 (50 ng), act5C-HA-rdx (0 or 500 ng), and UAS-Su(fu) (0 or 400 ng); for Figure 5E and S6, act5C-hh (500 ng), UAS-Ci-GFP (200 ng), act5C-GAL4 (100 ng), and act5C-HA-rdx (0 or 500 ng); for Figure 5C (right) and S5C, UAS-Ci-GFP (100 ng), act5C-GAL4 (100 ng), and act5C-HA-rdx (0 or 500 ng). The total amount of DNA was adjusted to 1.65 µg by adding the empty vector. Cells were observed 48 h after transfection. FRAP and FLIP analyses were performed using a 100× oil immersion objective, the 3× zoom on the Zeiss LSM510 confocal microscope and the 488 nm line of a Kr/Ar laser operating at 85% laser power and 2% transmission. The stage temperature was maintained at 25°C. For FRAP experiments, a specific region of the nucleus was selected and bleached using 100 iterations with 100% transmittance. Images were recorded at 1 sec intervals. Cells were imaged 50 times before photobleaching and 50 times afterwards, with maximum scan speed. For FLIP analysis, a region of the cytoplasm around the nucleus was selected and bleached by 50 iterations after imaging scans (10 times before photobleaching and 20 times after photobleaching). Images were acquired at 10 sec intervals. For every protein, or combination of proteins, at least 8 FLIP or FRAP curves were generated per transfection. For data analysis, the LSM software (Zeiss) was used to calculate the mean fluorescence intensity of the nuclear region. A fluorescence recovery curve was drawn using the Excel software, according to Rabut and Ellenberg [63]. The nuclear import rate of Ci-GFP was calculated as the slope of a linear regression fit of the initial phase of the flux curve (at least 5 post-bleach points).

### Western blotting and co-immunoprecipitation

To study the interaction of Importin α3 with Ci-155, clone-8 cells (4×10^6^ cells/6 cm dish) were transfected with the plasmid to express Hh (1 µg), HA-Ci-155 (1.5 µg), Myc-Importin α3 (1 µg), and 2xFlag-Rdx (1 µg for competition assay) using Cellfectin. Forty-eight hours after transfection, cells were lysed in 300 µl lysis buffer (50 mM HEPES (pH 7.4), 250 mM NaCl, 0.5% NP-40, 0.2 mM EDTA, protease inhibitor mixture). The lysates were diluted to 150 mM NaCl, and then immunoprecipitated with rat anti-HA (3F10) antibody or control normal rat IgG. The immunoprecipitates were analyzed by immunoblotting with either a rat or mouse anti-HA antibody, a mouse anti-Myc antibody and a mouse anti-Flag antibody.

### Importin α3 RNAi experiment

The *importin α3* cDNA was obtained from S. Cotterill [55]. PCR was performed using the following *importin α3* specific oligonucleotide primers: imp α3-F (forward) 5′–GGCCCTGGGCAACATCA–3′ and imp α3-R (reverse) 5′–CGCCTCCTTTCGGATCTT–3′, which contained a T7 polymerase binding site (TAATACGACTCACTATAGGG). Complementary single-stranded RNAs were generated using a Megascript T7 transcription kit (Ambion), and were annealed to form double-stranded RNA (dsRNA). Using Cellfectin, dsRNA was added to clone-8 cells at 5 or 10 µg/well, together with the expression vectors. Cells were used for each experiment 48 h after RNAi treatment.

## Supporting Information

Figure S1 **Rdx expression pattern in the wing disc.** (A) Expression of *rdx-lacZ* (green) was detected by X-gal staining. Anterior is to the left; dorsal is up. (B) Expression of *rdx-lacZ* (magenta) (b) and *hh>GFP* (green) (c) detected by imunostaining are superimposed in (a). Anterior is to the left; dorsal is up.
(TIF)

Figure S2 **Loss of Rdx expression did not upregulate** ***dpp*** **(A) and** ***ptc*** **(B) in the region close to the A/P boundary, where Rdx is highly expressed.** Clones of *rdx^11^* mutant cells marked by the absence of GFP (green) (a) in the wing pouch are superimposed with *dpp* expression monitored by the *dpp-lacZ* reporter (magenta) in (A-b) or with Ptc immunostaining (magenta) in (B-b). Clones of *rdx^11^* are surrounded by a white line (b). Upregulation of *dpp* was evident only in the *rdx^11^* clones away from the A/P boundary (indicated by arrow), while upregulation of *ptc* was not evident. (C) Schematic expression pattern of Rdx, *dpp* and *ptc* in the wing disc.
(TIF)

Figure S3 **Analysis of additional** ***rdx^11^*** **clones to demonstrate that loss of Rdx leads to the upregulation of** ***kn*** **.** As shown in Fig. 1C-c, d, clones of *rdx^11^* mutant cells were marked by the absence of the green GFP clonal signal (green), while expression of *kn* was monitored by the *kn-lacZ* reporter (magenta). Clones of *rdx^11^* are surrounded by a white line.
(TIF)

Figure S4 **Analysis of additional clones to demonstrate that Rdx does not induce Ci-155 degradation in the region close to the A/P boundary.** (A) As shown in Fig. 3A, clones of cells overexpressing Rdx marked by the presence of the GFP signal (green) (a) are superimposed with Ci-155 immunostaining (magenta) in (b). Rdx overexressing clones were surrounded by white line. (B) As shown in Fig. 3B, clones of *rdx^11^* mutant cells marked by the absence of GFP signal (green) (a) are superimposed with Ci-155 immunostaining (magenta) in (b). The A/P boundary and the boundary of the regions expressing low and high levels of Ci-155 are indicated by yellow lines. Clones of *rdx^11^* mutant were surrounded by white line.
(TIF)

Figure S5 **FRAP experiments.** (A) Nuclear Ci-GFP was photobleached, and then fluorescence recovery after photobleaching was monitored. Individual images at selected times are shown. Experiments were performed in the presence and absence of Hh (B and C, respectively) with or without Rdx.
(TIF)

Figure S6 **FLIP experiments.** (A) FLIP experiments were performed using clone-8 cells transfected with the Ci-GFP and Hh expression vector, in the presence or absence of the Rdx expression plasmid. (B) Individual images at selected times are shown.
(TIF)
