# Supplementary figures and images for: Inhibition of the Nuclear Import of Cubitus Interruptus by Roadkill in the Presence of Strong Hedgehog Signal
Source: PLoS One. 2010 Dec 15;5(12):e15365. doi: 10.1371/journal.pone.0015365 (PMC3002282; doi:10.1371/journal.pone.0015365)

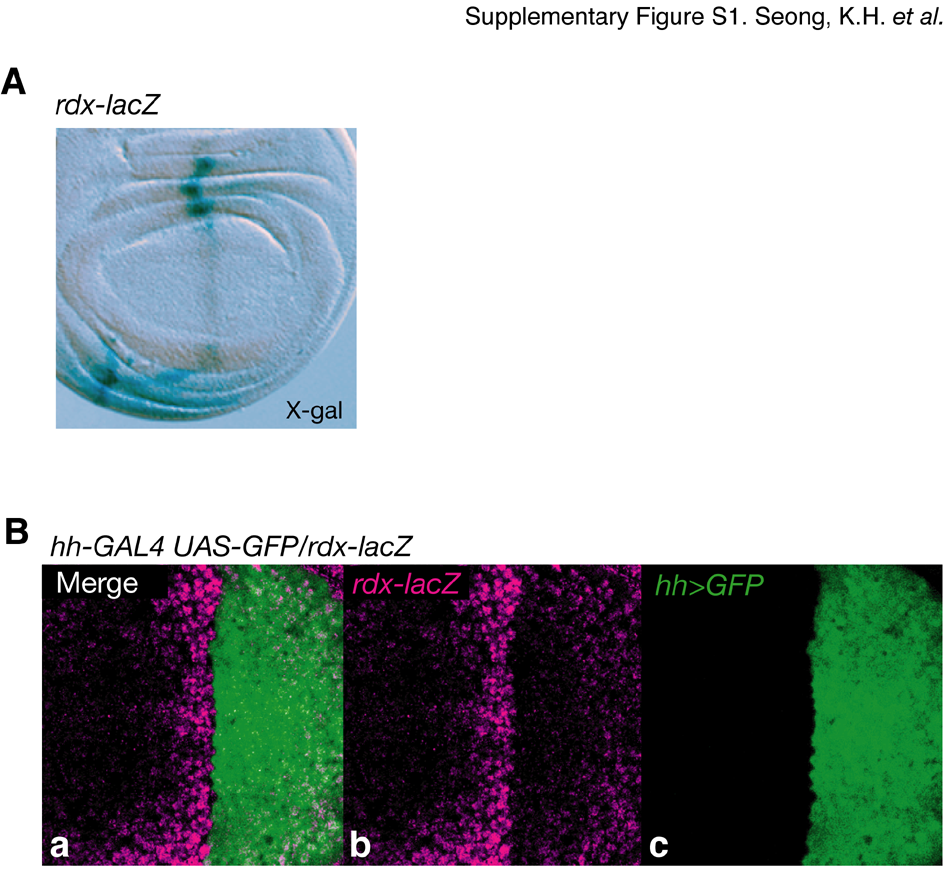

Supplement: Figure S1 — Rdx expression pattern in the wing disc. (A) Expression of rdx-lacZ (green) was detected by X-gal staining. Anterior is to the left; dorsal is up. (B) Expression of rdx-lacZ (magenta) (b) and hh>GFP (green) (c) detected by imunostaining are superimposed in (a). Anterior is to the left; dorsal is up. (TIF) [file pone.0015365.s001.tif]

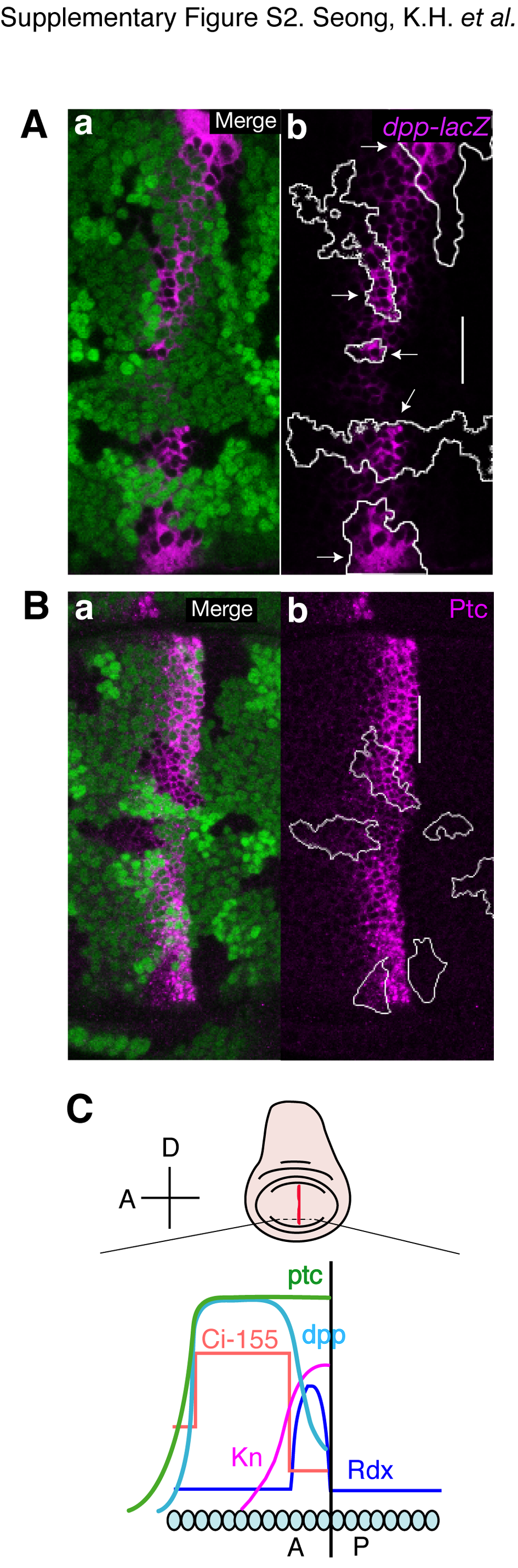

Supplement: Figure S2 — Loss of Rdx expression did not upregulate dpp (A) and ptc (B) in the region close to the A/P boundary, where Rdx is highly expressed. Clones of rdx11 mutant cells marked by the absence of GFP (green) (a) in the wing pouch are superimposed with dpp expression monitored by the dpp-lacZ reporter (magenta) in (A-b) or with Ptc immunostaining (magenta) in (B-b). Clones of rdx11 are surrounded by a white line (b). Upregulation of dpp was evident only in the rdx11 clones away from the A/P boundary (indicated by arrow), while upregulation of ptc was not evident. (C) Schematic expression pattern of Rdx, dpp and ptc in the wing disc. (TIF) [file pone.0015365.s002.tif]

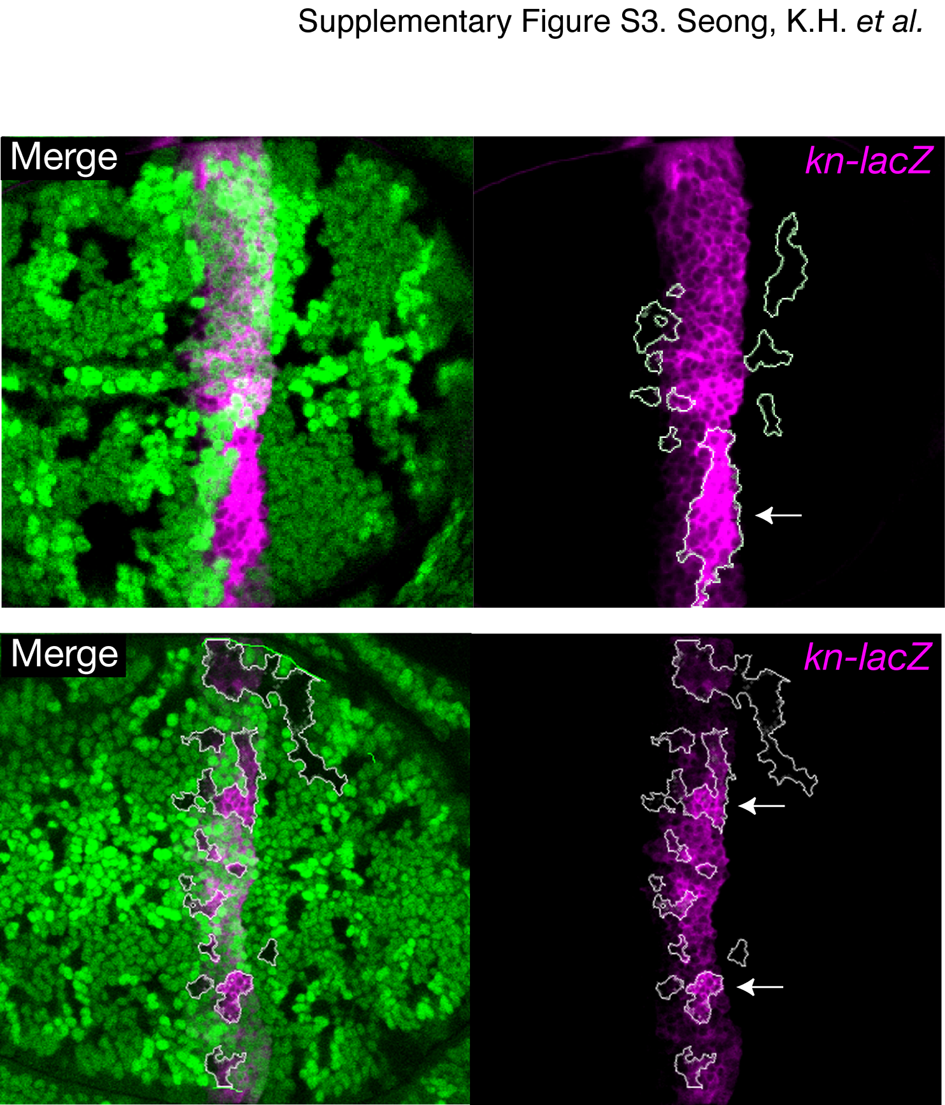

Supplement: Figure S3 — Analysis of additional rdx11 clones to demonstrate that loss of Rdx leads to the upregulation of kn . As shown in Fig. 1C-c, d, clones of rdx11 mutant cells were marked by the absence of the green GFP clonal signal (green), while expression of kn was monitored by the kn-lacZ reporter (magenta). Clones of rdx11 are surrounded by a white line. (TIF) [file pone.0015365.s003.tif]

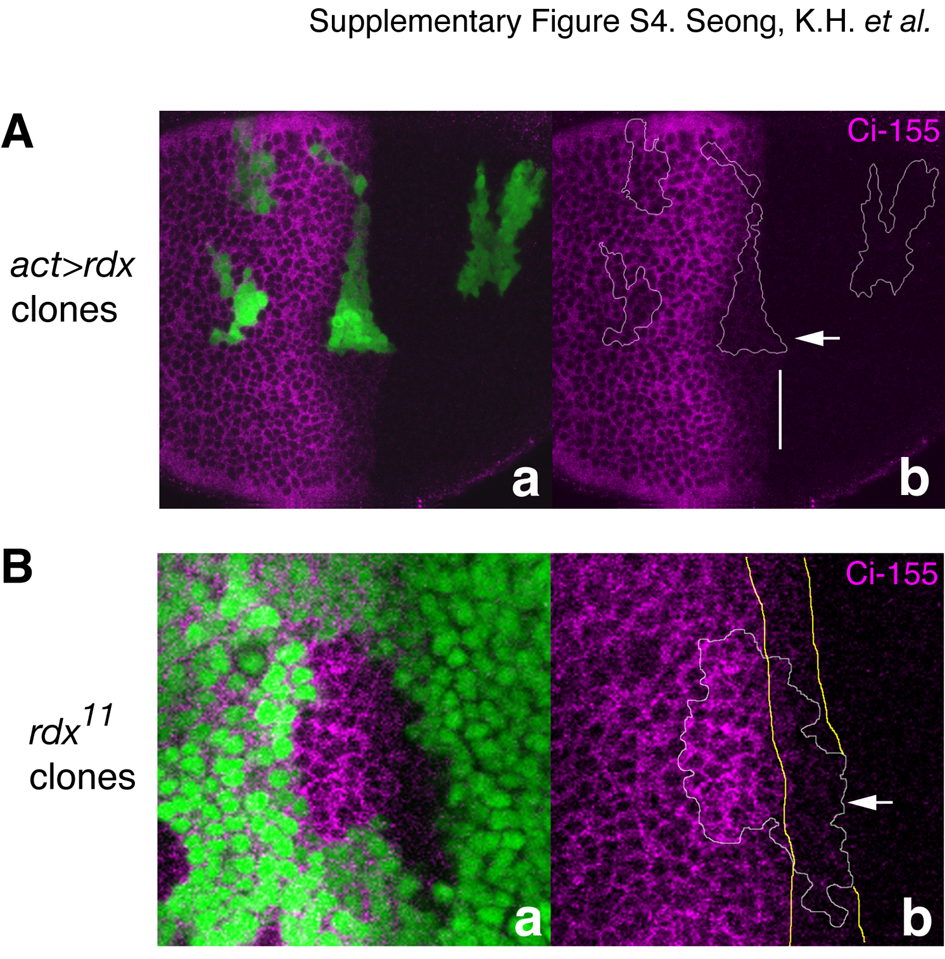

Supplement: Figure S4 — Analysis of additional clones to demonstrate that Rdx does not induce Ci-155 degradation in the region close to the A/P boundary. (A) As shown in Fig. 3A, clones of cells overexpressing Rdx marked by the presence of the GFP signal (green) (a) are superimposed with Ci-155 immunostaining (magenta) in (b). Rdx overexressing clones were surrounded by white line. (B) As shown in Fig. 3B, clones of rdx11 mutant cells marked by the absence of GFP signal (green) (a) are superimposed with Ci-155 immunostaining (magenta) in (b). The A/P boundary and the boundary of the regions expressing low and high levels of Ci-155 are indicated by yellow lines. Clones of rdx11 mutant were surrounded by white line. (TIF) [file pone.0015365.s004.tif]

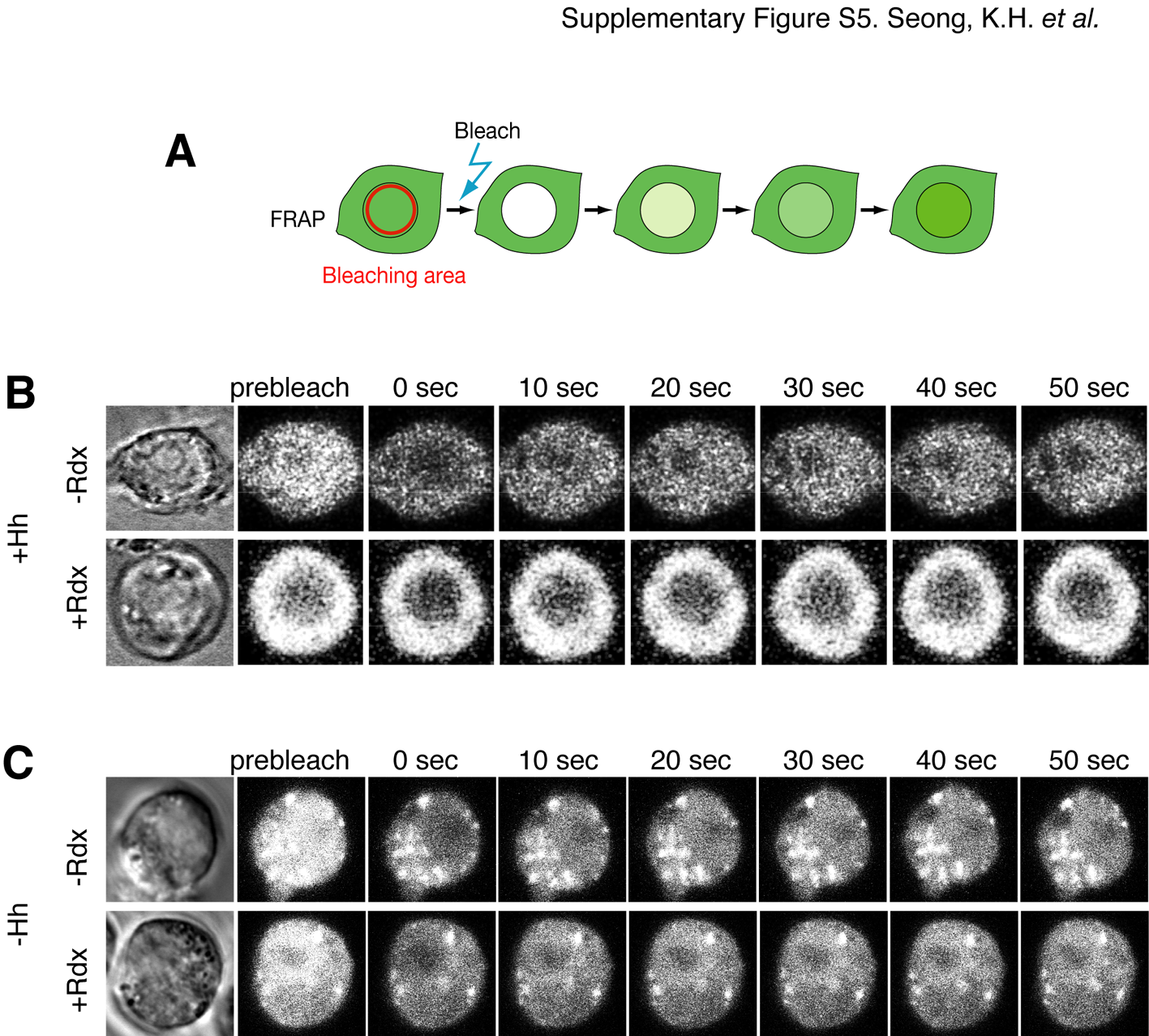

Supplement: Figure S5 — FRAP experiments. (A) Nuclear Ci-GFP was photobleached, and then fluorescence recovery after photobleaching was monitored. Individual images at selected times are shown. Experiments were performed in the presence and absence of Hh (B and C, respectively) with or without Rdx. (TIF) [file pone.0015365.s005.tif]

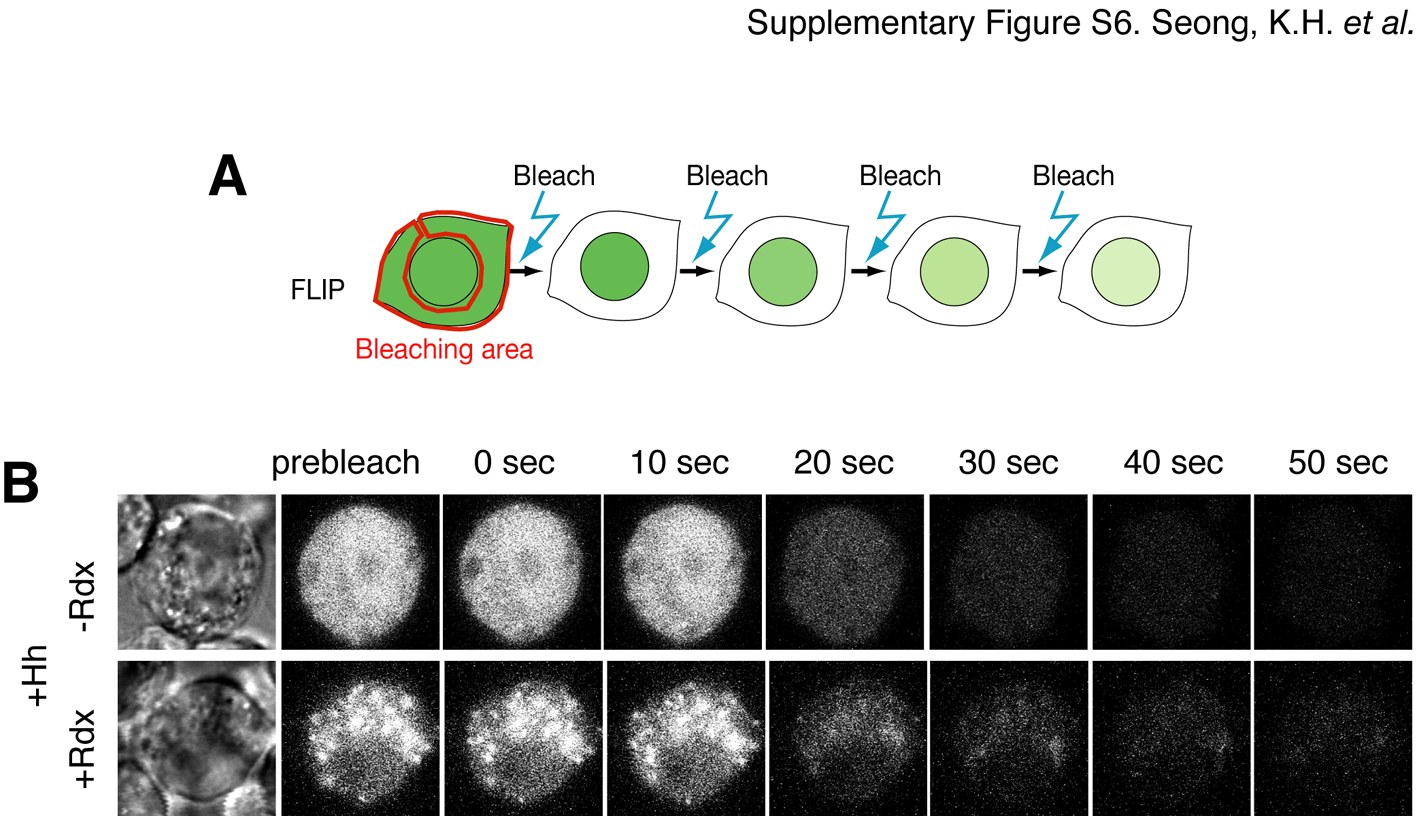

Supplement: Figure S6 — FLIP experiments. (A) FLIP experiments were performed using clone-8 cells transfected with the Ci-GFP and Hh expression vector, in the presence or absence of the Rdx expression plasmid. (B) Individual images at selected times are shown. (TIF) [file pone.0015365.s006.tif]
